# Genome sequence of *Cohnella* sp. M.A.Huq-80 isolated from pond-side soil located in Anseong, South Korea

**DOI:** 10.1128/mra.01103-25

**Published:** 2025-12-15

**Authors:** Md. Amdadul Huq, Md. Ful Mia, Md. Morshedul Alam

**Affiliations:** 1 Department of Life Sciences, College of BioNano Technology, Gachon University 65440, Seongnam, Republic of Korea; 2 Research and development (R&D), Popular Pharmaceuticals PLC, Dhaka, Bangladesh; 3 Department of Biochemistry and Microbiology, School of Health and Life Sciences, North South University 54495, Dhaka, Bangladesh; University of Wisconsin-Madison, Madison, Wisconsin, USA

**Keywords:** Genome sequence, *Cohnella *sp. M.A.Huq-80, pond-side soil

## Abstract

This study reports the draft genome sequence of *Cohnella* sp. M.A.Huq-80, a bacterial strain isolated from the soil sample of a pond located in Anseong, South Korea, in 2021. The genome consists of 6,429,982 base pairs assembled into 45 contigs, encoding 5,416 predicted protein coding genes. Members of the genus *Cohnella* are significant for their roles in soil ecology and for producing thermostable enzymes valuable in biotechnology and biomass degradation.

## ANNOUNCEMENT

The genus *Cohnella*, first proposed by Kämpfer et al. (1), belongs to the family *Paenibacillaceae*, which currently comprises 36 recognized species with validly published names (2) that have been isolated from different environments, including pond, root nodules, soils, freshwater, and wetland (3). During investigations on bacterial biodiversity in the soil of a pond, a new strain of the genus *Cohnella*, designated *Cohnella* sp. M.A.Huq-80 was isolated, and its draft genome assembly is presented in this study. The genus *Cohnella* is significant for its ecological roles and production of industrially relevant thermostable enzymes while also contributing to microbial diversity research and offering potential applications in bioremediation and plant–microbe interactions (1, 4–6).

Strain M.A.Huq-80 was isolated from the soil sample of a pond, located in Anseong, South Korea. One gram of soil sample was suspended in 9 mL of sterile 0.85% (w/v) NaCl solution (7). The suspension was serially diluted up to a 10^−6^ dilution, and 100 µL of individual dilution was spread onto TSA plates. Then, the plates were placed at a 28°C incubator for 3 days. Single colonies were purified by repeated streaking on fresh TSA plates (8). The strain M.A.Huq-80 has been deposited to China General Microbiological Culture Collection Center with deposition number CGMCC 1.60091. The genomic DNA was extracted from the bacterial culture that was grown in TSB for 24 hours using Solg Genomic DNA Prep kit (Solgent, Republic of Korea), according to the manufacturer’s protocol. The quality and concentration of the extracted genomic DNA were assessed using a NanoDrop 2000 spectrophotometer (Thermo Fisher Scientific, USA). The DNA fragments were size selected using AMPure XP beads to isolate those within the desired range, typically 200–300 bp for standard Illumina sequencing libraries. The library for sequencing was prepared using the standard library preparation kit for Illumina sequencing. After library preparation, sequencing was carried out using the Illumina HiSeq 3000 platform with paired-end 2 × 150 bp reads following the standard protocol provided by the manufacturer. Illumina’s bcl2fastq software version 2.20.0 was utilized with default parameters to demultiplex the raw reads and convert them into FASTQ format. Reads were processed with Trimmomatic version 0.38 (9). The genome sequence was assembled by SOAPdenovo v2.04 assembler (10). The genome was annotated with the NCBI Prokaryotic Genome Annotation Pipeline (PGAP) version 6.3 (11–13). The strain was identified using a whole genome-based taxonomic analysis in the Type (Strain) Genome Server (14). To estimate the degree of pairwise relatedness between M.A.Huq-80 and the closest type strain, BLAST-based average nucleotide identity (ANI) was calculated (15). The best matches to the strain M.A.Huq-80 genome are the type strain *Cohnella yongneupensis* KACC 11768 (accession GCA_042655785) with ANI value of 79.77%.

The genome of *Cohnella* sp. M.A.Huq-80 is 6,429,982 bp long with a GC content of 58.0%. The assembly contains 45 contigs with a coverage of 53×. A total of 5,518 genes were predicted, of which 5,416 are protein-coding genes. The details of genomic features are presented in Table 1.

**TABLE 1:** Genomic features of *Cohnella* sp. M.A.Huq-80

Description of source	
Location	Anseong, South Korea
Time	2021
Type	Pond-side soil
Sequencing summary	
Coverage	53×
Total bases	6,429,982
GC content	58.0%
Assembly report	
Contigs	45
Contig *L*_50_	5
Contig *N*_50_	340 kb
Genome length	6.4 Mb
Annotation report	
Genes (total)	5,518
CDSs (total)	5,443
CDSs (with protein)	5,416
ncRNAs	4
tRNA	56

## Data Availability

The draft genome sequence of *Cohnella* sp. M.A.Huq-80 was deposited in GenBank under accession number JBQWTP000000000. The raw reads are available in SRA under accession number SRR33604985. The version described in this paper is the first version, JBQWTP000000000.1.
